# Sub-Diaphragmatic Abscess

**Published:** 1900-10-13

**Authors:** 


### SUB-DIAPHRAGMATIC ABSCESS.

The space below the diaphragm is one which is not easy to get at, either for diagnosis or for treatment, and when an abscess forms there it becomes a question of serious urgency to determine how to give relief.

A good review of the whole subject of sub-diaphragmatic abscess was given at the Ipswich meeting of the British Medical Association. After giving a long list of the causes by which abscess in this region might be produced, Mr. Rickman Godlee pointed out the important fact that subphrenic abscess is not an isolated disease occurring on its own account, but is almost of necessity a complication of something else. It is therefore by no means easy to isolate the symptoms due to it, and say that such and such are the symptoms by which its presence can be diagnosed.

Being an accident or by-product of some other disease, the symptoms of the latter will probably be by far the most prominent ones, and may by their aggressiveness lead to the abscess being overlooked. Say, for example, that the disease starts as an appendicitis, and that the lump in the groin subsides as the abscess tracks up outside the ascending colon, and spreads out under the diaphragm, how easily it may be overlooked or mistaken for a secondary pleural effusion.

To show how impossible it is to lay down any definite series of symptoms as indicating without doubt the presence of sub-diaphragmatic abscess, one has but to point to the variety in shape and size which such an abscess may assume, the very different positions it may occupy, and the very numerous diseases by which it may be caused. Such abscesses may be divided primarily into extraperitoneal, as when they start from caries of the spine or ribs, or spread up from the kidneys, and intraperitoneal, which is by far the commoner form, when they start from the rupture of an abscess in one of the viscera below the diaphragm. "Think only," said Mr. Godlee, "of such a case as that of a very chronic ulcer of the stomach which has caused extensive adhesions between the viscus and the diaphragm; an abscess may form amongst these adhesions and burrow about by complicated and narrow tracks until it points perhaps through an intercostal space. Then contrast with it one in which a sudden perforation of a stomach ulcer has occurred, but in which the suppuration is to some extent localised and contains air. It is difficult to trace a family resemblance between these two conditions."

Subphrenic abscess may be defined as any collection of pus, or of pus and gas, the whole or part of which intervenes between the diaphragm and the structures normally in contact with it. The great point in regard to diagnosis is to separate the disease from pleurisy, from which, however, it ought to be distinguishable by the following points:—

(a) The movements of the chest are not impaired on the affected side.

(b) The upper limit of dulness is not so sharply defined.

(c) Breath sounds may be heard below the level of the dulness, and if a deep inspiration be made the line at which the breath sounds and vocal resonance are heard, and at which vocal fremitus is felt, is distinctly lowered.

(d) When there is much emaciation the lower border of the lung may sometimes be distinctly seen moving up and down during respiration, showing clearly that the cause of the dulness is below and not above the diaphragm.

Instead of dulness there may be a great extent of tympanitic resonance, and over the resonant area the "bell sound" may be elicited in a most perfect manner from the presence of gas as well as pus. From the similarity of the signs to those of pneumothorax this condition has been called "subdiaphragmatic pneumothorax," and it is surprising how high this tympanitic area (or the dull area for the matter of that) may extend, rising up as high as the second rib and reaching down into the iliac fossa.

Among the important points raised in the discussion may be mentioned one made by Mr. Bidwell, to the effect that a peculiarly offensive character in the pus withdrawn from an empyæma, and the fact that an empyæma does not improve after resection of the rib and irrigation, should make one suspect the presence of subdiaphragmatic abscess; and one made by Mr. Bruce Clarke, who pointed out how great an aid to diagnosis was invariably given in cases of doubt by introducing the hand into the peritoneal cavity and investigating fully the abnormal condition.

